# Operations for Removal of the Kidney

**Published:** 1883-03

**Authors:** 


Operations for removal of the kidney are not infrequent, nor are they dangerous. Recently after extirpation of one kidney the patient died, and an autopsy revealed that a fellow-kidney had never existed.

In this number of the Independent Practitioner will be found an account of a somewhat similar operation : the patient suffered from hydronephrotic distension of one kidney, and, death occurring with uræmic symptoms, an autopsy revealed the fellow-kidney to be in an advanced stage of chronic Bright's, incapable of performing excretory functions necessary to life.

We read this article shortly after hearing of the case first referred to, and the thought forced itself upon us, how can unilateral disease or abnormity of the kidney be diagnosticated during life ?

There are several methods of preventing fluids from passing from one reservoir into another through flexible tubing; plugging of either orifice and pressure upon the duct sufficient to occlude it are, of course, the two that at once suggest themselves.

Can we plug or tie, or press on a ureter so as to let the bladder fill with urine from the kidney on the other side? If so, we can diagnosticate unilateral disease of the kidney, if it exist, and if urinary symptoms count for anything.

The absence of a kidney would, we opine, be a much more difficult matter to determine. Besides, if only one kidney existed and its ureter were plugged or ligated, uræmic symptoms might ensue.

The whole matter is one of the greatest importance, both on account of the (comparative) frequency with which nephrectomy is performed and the paucity of literature and experience in matters concerning so delicate and intricate a task as diagnosis of abnormities in an organ, the manifestation of which can so readily be masked or altered.

